# Mesenchymal Stem Cell Therapy for Superior Gluteal Nerve Injury Post-hip Arthroplasty: A Case Report

**DOI:** 10.7759/cureus.79088

**Published:** 2025-02-16

**Authors:** Roberto D Rached, Thomas Helfenstein, Angela H Kim, Thadeu R Da Costa, Ricardo L Araújo

**Affiliations:** 1 Physical Medicine and Rehabilitation, Hospital das Clínicas da Faculdade de Medicina da Universidade de São Paulo, São Paulo, BRA

**Keywords:** axonal injury, functionality isokinetic, mesenchymal stem cells (mscs), musculoskeletal rehabilitation, nerve regeneration

## Abstract

Peripheral nerve injuries, such as axonal injury of the superior gluteal nerve, are rare but debilitating complications that can occur after hip arthroplasty. This case report describes the use of adipose-derived mesenchymal stem cells (MSCs) to treat an axonal injury of the superior gluteal nerve in a 71-year-old patient. After conventional rehabilitation failed, MSC infiltration was chosen and performed with ultrasound (US) guidance. Two months later, the patient showed normalization of electromyography (EMG), indicating full nerve recovery, along with significant improvement in neuropathic pain. The patient also demonstrated a 55% increase in maximum torque and a 9% increase in power during right hip extension in isokinetic evaluation, resulting in improvement of muscle strength and functionality. This case highlights the potential of MSCs in promoting nerve regeneration, suggesting that this approach may accelerate nerve recovery and improve short-term clinical outcomes. Although the results are promising, further studies are needed to confirm the efficacy and safety of this treatment in a larger population. This integrated model of cell therapy and physical rehabilitation represents a significant advance in recovering from complex nerve injuries.

## Introduction

Peripheral nerve injuries, such as axonal injury of the superior gluteal nerve, are rare but debilitating complications that can occur after surgical procedures like hip arthroplasty [1]. These injuries often result in significant motor deficits, neuropathic pain, and functional impairment, negatively affecting the patient’s quality of life [1]. Spontaneous regeneration of peripheral nerves is limited, and traditionally, therapeutic options for such injuries have shown variable, often unsatisfactory outcomes.

In recent years, the use of mesenchymal stem cells (MSCs) has emerged as a promising approach in regenerative medicine, with the potential to improve nerve injury recovery. MSCs, derived from various sources such as bone marrow, adipose tissue, and umbilical cord, possess immunomodulatory, anti-inflammatory, and neurotrophic factor secretion properties that may promote neuroprotection and axonal regeneration [2].

Although scientific literature has documented the beneficial effects of MSCs in animal models of nerve injury, clinical data in humans remains limited, particularly concerning nerve injuries associated with orthopedic surgeries [2,3]. This case report presents the application of adipose-derived mesenchymal cells in the treatment of an axonal injury of the superior gluteal nerve following hip arthroplasty, highlighting the methodology employed, observed clinical outcomes, and implications for future therapeutic approaches.

This case aims to contribute to the growing evidence base regarding the use of mesenchymal cells in peripheral nerve injuries, suggesting that this therapy may offer new hope for patients with nerve damage associated with complex surgical procedures. Objective parameters such as the visual analog scale (VAS) for pain, electromyography, and an isokinetic device (Cybex, USA) were used to assess improvements in pain, neuromuscular conduction of the superior gluteal nerve, and muscle strength and power in the hip muscles, respectively. It represents an innovative approach that could serve as a crucial bridge to effective rehabilitation, significantly reducing the time required for functional recovery.

## Case presentation

The patient, DMB, a 71-year-old female with a history of right hip osteoarthritis for over five years, underwent total hip arthroplasty in January 2023. She developed a complication, with a collection seen on computed tomography, accompanied by burning neuropathic pain rated 10/10 on the VAS, as well as reduced strength and sensitivity in the gluteal region. A second surgical intervention was performed 20 days later, involving the exchange of femoral stem material and surgical debridement. Methicillin-resistant *Staphylococcus aureus* (MRSA) was detected in soft tissue cultures, and the patient was prescribed a three-month course of antibiotics (Teicoplanin 400 mg IV every 24 hours and Rifampin 300 mg orally every 12 hours). The patient started a rehabilitation program involving motor physiotherapy and physical conditioning but continued to experience significant difficulty in walking and performing transfers, relying on assistive devices, and requiring assistance with basic activities of daily living (ADLs).

After more than a year of rehabilitation without satisfactory results, a post-operative complication investigation was initiated. EMG in April 2024 revealed axonal injury of the superior gluteal nerve, with normal sensory-motor neuroconduction in the evaluated segments but signs of active denervation (positive sharp waves) and recent partial reinnervation (long-duration, high-amplitude polyphasic potentials) in the gluteus medius and minimus muscles. Given the patient's disability and episodes of neuropathic pain rated at 10/10 on the VAS, along with the failure of conventional rehabilitation, MSC treatment was chosen.

The procedure was performed on June 11, 2024, under strict aseptic conditions to minimize infection risk and ensure patient safety. Initially, a transverse abdominis plane (TAP) block was performed to provide effective analgesia and reduce the need for systemic anesthetics during the procedure. After the anesthetic block, a Klein solution, containing lidocaine, epinephrine, and saline, was injected into the infraumbilical adipose tissue area for liposuction. Adipose tissue was aspirated using a specific cannula and processed to obtain mesenchymal cells. The collected material was emulsified to an appropriate consistency (grade 2.4) to maximize viable cell concentration. The emulsified material was then filtered to remove larger particles and unwanted residues, resulting in a cell suspension suitable for infiltration.

**Figure 1:**
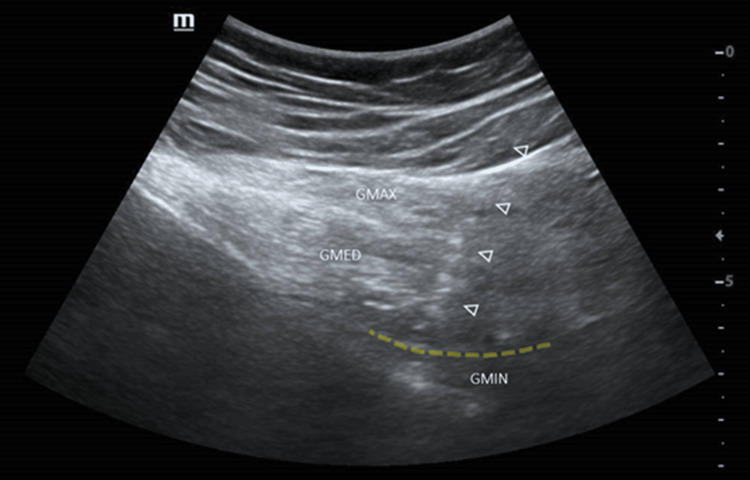
Ultrasound image demonstrating the infiltration of MSCs into the fascia between the gluteus minimus and gluteus medius Arrowheads: needle; dotted yellow line: fascia between the GMIN and GMED GMIN: gluteus minimus; GMED: gluteus medius; GMAX: gluteus maximus

The infiltration of MSC was performed following a standardized technique to ensure reproducibility and safety. The cells used were autologous, obtained from adipose tissue, and processed to ensure viability and sterility. MSC infiltration was performed directly at the site of the superior gluteal nerve injury, in the plane between the gluteus medius and minimus muscles (Figure 1). The procedure was guided by ultrasound (US) (DC 8 Mindray, Mindray Medical International Limited, China), ensuring precise application of the cells exactly at the affected region, maximizing the chances of nerve regeneration. US guidance also allowed real-time visualization of the cell material's distribution, ensuring that the injured area was fully covered. The use of the TAP block contributed to a more comfortable immediate postoperative period, facilitating pain management and enabling smoother recovery. This combined approach of advanced regional anesthesia techniques, careful cell preparation, and precise image-guided infiltration exemplifies the complexity and sophistication involved in treating nerve injuries with mesenchymal cells.

Sixty days after the intervention, a new EMG showed complete recovery of the axonal injury, with no abnormalities in neuroconduction or EMG in the segments evaluated, including the gluteus medius and minimus muscles innervated by the superior gluteal nerve. Additionally, the intervention served as a catalyst for the entire rehabilitation process. During this period, the patient also experienced significant improvement in neuropathic pain, now reduced to 3/10 on the VAS.

**Figure 2:**
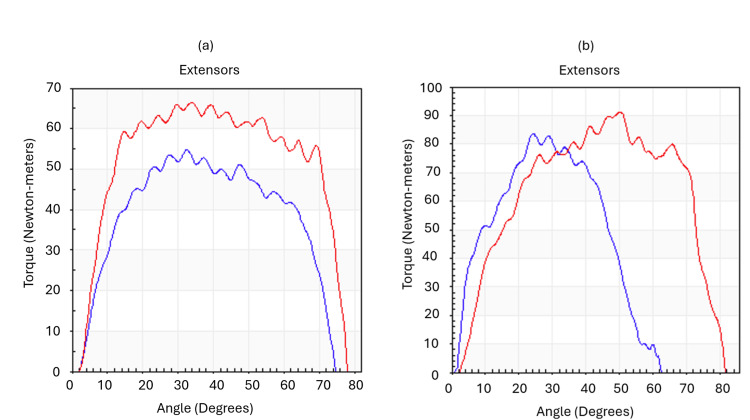
Isokinetic graphics of hip extension: torque (Newton-meters) × angle (degrees) Graph (a) was performed before MSC infiltration on April 11, 2024, and graph (b) was performed after the infiltration on August 26, 2024. The blue lines refer to the right hip and the red lines refer to the left hip. MSC: mesenchymal stem cell

The Cybex, an isokinetic device, was used as an objective evaluation tool. This device measures muscle strength at different angular velocities, allowing precise analysis of parameters such as maximum torque, power, and total work [4]. In this case report, the patient showed significant improvement after two months of intervention, with a 55% increase in maximum torque and a 9% increase in maximum power during right hip extension, compared to the first examination conducted in April 2024 (Figure 2).

These gains indicate considerable improvement in muscle strength and functionality, essential for daily activities. Although there was a reduction of 33 Joules in total work, this decrease does not compromise the interpretation of the results, as the patient was able to perform less total work (less total energy generated) due to improved muscle efficiency. Furthermore, during right hip flexion, a 24% increase in maximum torque and a 93% increase in maximum power were observed, along with an increase of 58 Joules in total work, representing a substantial improvement in the overall function of the right hip.

Appropriate permission for publication was obtained from the patient featured in the article, who signed the informed consent form and is currently under medical follow-up.

## Discussion

This case report highlights the use of MSCs as an innovative therapeutic approach for nerve regeneration in a patient with axonal injury of the superior gluteal nerve following hip arthroplasty. The observed recovery, both in terms of neuromuscular function and quality of life, supports the growing evidence that MSCs play a significant role in promoting neuroregeneration [3,5]. The use of MSCs for nerve damage repair has been extensively explored in research involving rats, yielding excellent results. However, in humans, studies remain unstandardized [6].

The initial study suggesting the use of MSCs for neuronal regeneration was published in 2005. Since then, various animal and human models have been developed to investigate its therapeutic possibilities [7]. Research has extensively explored their potential applications in multiple fields, such as ophthalmology (for optic nerve regeneration), urology (for treating nerve-related erectile dysfunction), and reconstructive plastic surgery [8-10].

In peripheral nerve injuries, clinical outcomes after nerve damage are often poor, though some regeneration may occur if an intrinsic adaptive response is induced. However, axons generally exhibit slow regrowth and inadequate reinnervation of the target muscle. Regenerative capacity diminishes over time due to prolonged axotomy and denervation of Schwann cells [11]. In this case, two months after the MSC application, the patient showed surprisingly positive results. The EMG, which previously indicated significant axonal injury, returned to normal, signifying restored nerve conduction and functional integrity of the superior gluteal nerve. This finding suggests accelerated nerve recovery, potentially facilitated by the regenerative properties of MSCs, which include the secretion of neurotrophic factors and modulation of the inflammatory response.

MSCs secrete various growth factors such as brain-derived neurotrophic factor (BDNF), nerve growth factor (NGF), and glial cell line-derived neurotrophic factor (GDNF) [ [3,5]. These factors are crucial for neuron survival, promoting axonal regeneration, and the formation of new synapses. This neurotrophic support stimulates the recovery of nerve function by facilitating the growth of damaged axons and improving communication between neurons and muscles [2,3]. Furthermore, MSCs release anti-inflammatory cytokines such as IL-10 and transforming growth factor-beta (TGF-β), which suppress aggressive immune cell activity, reducing inflammation and minimizing secondary tissue damage [5]. This creates a more favorable environment for axonal regeneration and limits the impact of chronic inflammation, which could otherwise delay the repair process [5].

Additionally, MSCs promote angiogenesis by releasing factors such as vascular endothelial growth factor (VEGF) [12,13]. Angiogenesis improves tissue perfusion in the injured nerve, ensuring adequate oxygen and nutrient supply [14]. MSCs may also prevent the formation of glial scars, which can serve as a physical barrier to axonal regeneration after nerve injury [13]. These physiological mechanisms render MSCs a powerful tool in nerve regeneration, offering multifaceted support that accelerates the functional recovery process after axonal injury.

In addition to the electrophysiological improvements, substantial progress was observed in the muscle strength of the gluteus medius and minimus, both innervated by the superior gluteal nerve. With increases in maximum torque and power, evidenced by isokinetic evaluation, the patient was performing each contraction more efficiently, concentrating more energy into fewer repetitions. This may explain the lower total work, despite higher quality in strength and power. The patient reported progressive recovery of strength, leading to greater pelvic stability during ambulation. The improvement in walking was clinically significant, with the patient regaining a normal gait pattern without claudication or significant pain.

Overall functionality also improved considerably, as demonstrated by standardized functional assessments and subjective reports of enhanced ability to perform daily activities. This improvement in functionality was reflected in a better quality of life, with the patient reporting increased independence and reduced emotional and psychological distress related to her previous condition. These results are encouraging and reinforce the potential of MSCs as a therapeutic intervention for peripheral nerve injuries. The early normalization of EMG findings, combined with functional recovery and enhanced quality of life, suggests that MSC treatment not only accelerates nerve regeneration but also improves short-term clinical outcomes.

However, despite these promising results, it is important to recognize that this is a case report, and therefore, conclusions should be interpreted cautiously. Additional studies, including controlled clinical trials, are necessary to confirm the efficacy and safety of this approach in a larger population of patients. Furthermore, long-term follow-up will be essential to assess the durability of therapeutic effects and to identify any potential late complications.

## Conclusions

This case report contributes to the growing evidence that MSCs may be a powerful tool in nerve regeneration, offering new hope for patients with nerve injuries associated with complex surgical procedures. The combination of cell therapy and physical rehabilitation can, therefore, provide a more complete and faster recovery, improving patients’ quality of life and reducing long-term functional limitations. This integrated treatment model represents a significant advance in regenerative and rehabilitative medicine, suggesting a promising future for the recovery of complex nerve injuries.
